# Cost-effectiveness analysis for SilAtro-5-90 adjuvant treatment in the management of recurrent tonsillitis, compared with usual care only

**DOI:** 10.1186/s12962-021-00313-4

**Published:** 2021-09-19

**Authors:** Thomas Ostermann, A-La Park, Sabine De Jaegere, Katharina Fetz, Petra Klement, Christa Raak, David McDaid

**Affiliations:** 1 Department of Psychology and Psychotherapy, Witten/Herdecke University, Alfred-Herrhausen-Straße 50, 58448 Witten, Germany; 2 Care Policy and Evaluation Centre, Department of Health Policy, London School of Economics and Political Science, Houghton Street, London, WC2A 2AE UK; 3 Deutsche Homöopathie-Union, DHU-Arzneimittel GmbH & Co. KG, Ottostraße 24, 76227 Karlsruhe, Germany; 4 Institute of Integrative Medicine, Witten/Herdecke University, Gerhard-Kienle-Weg 4, 58313 Herdecke, Germany

**Keywords:** Tonsillitis, Homeopathy, Cost effectiveness, Acute throat infections

## Abstract

**Purpose:**

Antibiotics are one possible treatment for patients with recurrent acute throat infections (ATI), but effectiveness can be modest. In view of worries over antibiotic resistance, treatment pathways that reduce recurrence of ATI are essential from a public health perspective. Integrative treatment strategies can be an option but there is still a high demand to provide evidence of their cost effectiveness.

**Methods:**

We constructed a 4-state Markov model to compare the cost-effectiveness of SilAtro-5-90 as adjuvant homeopathic therapy to care as usual with care as usual alone in reducing the recurrence of ATI for children and adults with suspected moderate recurrent tonsillitis. The analysis was performed from a societal perspective in Germany over a 2-year period. Results are reported separately for children < 12 and for individuals aged 12 and over. The model draws on evidence from a multi-centre randomised clinical trial that found this strategy effective in reducing recurrence of ATI. Costs in 2019 € and outcomes after 1 year are discounted at a rate of 3% per annum.

**Results:**

For adults and adolescents aged 12 years and over, incremental cost per ATI averted in the adjuvant therapy group was €156.64. If individuals enter the model on average with a history of 3.33 previous ATIs, adjuvant therapy has both lower costs and better outcomes than care as usual. For children (< 12 years) adjuvant therapy had both lower costs and ATI than care as usual. The economic case is stronger if adjuvant treatment reduces surgical referral. At a hypothetical cost per ATI averted threshold of €1000 probabilistic sensitivity analysis suggests Silatro-5-90 has a 65% (adults) and 71% (children) chance of being cost-effective.

**Conclusion:**

Our results indicate the importance of considering homeopathy as adjuvant therapy in the treatment of ATIs in individuals with recurrent tonsillitis from a socio-economic perspective. Further evaluation should assess how differences in uptake and sustained use of homeopathic adjuvant therapy, as well as changing patterns of antibiotic prescribing, impact on cost effectiveness.

**Supplementary Information:**

The online version contains supplementary material available at 10.1186/s12962-021-00313-4.

## Introduction

Respiratory tract infections (RTIs) are quite common reasons to consult a general practitioner (GP) and according to epidemiological data account for 75% of antibiotic prescriptions written in the US [1, 2]. Tonsillitis is one major category of RTIs that is indicated by a swelling and inflammation of the pharyngeal tonsils and the back of the throat, including the adenoids and the lingual tonsils. Other signs and symptoms of tonsillitis include a sore throat and difficulty in swallowing. In acute tonsillitis, which is mostly of viral origin, infections may initially respond to symptomatic drugs (e.g., analgesics) and antibiotic therapy but may return episodically leading to a diagnosis of recurrent tonsillitis [2]. In both acute and recurrent tonsillitis, health professionals are conscious of the risks of antimicrobial resistance (AMR) that can result from excess use of antibiotics. In order to cope with AMR, a “no antibiotics” or a “delayed antibiotics” strategy is suggested, while taking specific clinical criteria into account [3, 4].

When applying this strategy, the use of homeopathic remedies could be part of the solution [5]. Homeopathy was developed by the German physician Samuel Hahnemann 200 years ago. It is based on ‘the principles of similars’ and ‘like cures like’ saying that diluted and potentized substances that cause symptoms in healthy individuals are used to treat patients with similar symptoms [6]. Homeopathy is geographically widespread and is the fourth most frequently used complementary and alternative medicine (CAM) therapy [7]. Advantages of this therapy, explaining its popularity, may include fewer and less serious side effects [8, 9].

Any treatment strategy that may help reduce the need for antibiotic use potentially is of great public health interest and help to face the growing threat of AMR [10]. The reduction of acute throat infections (ATI) and the need for medication may also have an impact on resource use and costs within health systems and for society. It could mean less time out of education and work.

Some studies have shown the effectiveness of homeopathic treatment for patients with these conditions. In an early study, Wiesenauer reported positive results in symptom reduction in an observational study of 107 patients that were treated homeopathically without antibiotics [11]. In another randomised, double-blind, placebo-controlled, 6-day pilot study in 30 children in South Africa, the homeopathic group had a statistically significant reduction in pain, inflammation of the pharynx and tonsil size [12]. However, the evidence to date has been equivocal with a recent meta-analysis of 8 trials comparing oral homeopathic treatment as an alternative to either placebo or standard treatment to prevent or treat RTIs in children reporting limited benefits [13]. However, according to a recent narrative review on the contribution of Integrative Medicine to reduce antibiotic use there is a high demand to evaluate integrative treatment strategies that may contribute to reducing antibiotic use and to providing evidence of their cost-effectiveness [14].

In this study, we consider the use of homeopathic medicine as an adjuvant rather than as an alternative treatment for recurrent throat infections. For example, a recent randomised controlled trial reported significantly better outcomes for adjuvant homeopathic therapy in the treatment of pre-adolescent children with upper respiratory tract infections and fever [15]. Another promising intervention that has been used for the treatment of patients aged 6–60 experiencing tonsillitis is the complex homeopathic medicine SilAtro-5-90. A multicentre randomised clinical trial of patients experiencing recurrent tonsillitis found that the risk of having an ATI was significantly lower in patients treated with conventional symptomatic medication together with SilAtro-5-90 (intervention group) than in those treated with conventional symptomatic medication alone (usual care group). At the same time, the adjuvant SilAtro-5-90 plus usual care group had a significantly reduced use of antibiotic medication, and a lower number of days absent from work or school when experiencing an ATI [16].

Based on these findings, the authors suggested further research to investigate the possible cost-effectiveness of SilAtro-5-90 in the treatment of recurrent tonsillitis. This paper thus explores whether adjuvant homeopathic medicinal treatment with SilAtro-5-90 alongside usual care in the treatment of recurrent tonsillitis is cost-effective by means of an economic modelling analysis (see Additional file 1 for a detailed description).

## Method

### Model overview and interventions

A 4-state Markov model has been constructed to assess the cost-effectiveness of SilAtro-5-90 as an adjuvant homeopathic therapy to care as usual compared with care as usual alone in reducing the recurrence of ATIs for a hypothetical population of children and adults with suspected moderate recurrent tonsillitis in Germany over a 2-year period. The primary outcome for the model is the incremental cost per ATI averted. Estimates of the recurrence of ATIs following intervention are drawn from an open-label multi-centre randomised controlled trial of SilAtro-5-90 [16] for individuals with moderate recurrent tonsillitis in 19 study centres across Germany, Spain and the Ukraine. Moderate recurrent tonsillitis was defined as experiencing at least 3 ATIs in the prior 12 months or 2 ATIs in each of the previous 2 years.

In that study 256 individuals aged 6–60 were randomised to SilAtro-5-90 plus usual care compared to usual care alone. The adjuvant intervention, SilAtro-5-90 (Tonsilotren^®^, Deutsche Homöopathie-Union, DHU-Arzneimittel GmbH & Co., KG), is a complex homeopathic medicinal product containing 5 active ingredients in different potencies (from D2 to D8 with D2 corresponding to a 1:10^2^ and D8 a 1:10^8^ dilution). While Atropinum sulfuricum (D5) and Mercurius bijodatus (D8) are well proven to treat the acute phase of the tonsillitis with typical symptoms such as dark red throat and difficulties in swallowing, and Hepar sulfuris (D3) is helpful in the treatment of impending suppuration, Kalium bichromicum (D4) is more used for subacute course of inflammation with thick yellowish discharges [17, 18]. The final active ingredient Silicea (D2) is a commonly used homeopathic remedy in the treatment of chronic diseases, which stimulates the regeneration of tissues and thus improves healing in chronic processes [17]. In the trial, there were 3 treatment periods, each of 8 weeks, spread uniformly across the 12-month trial. After each period of treatment, there was an 8-week observation period without adjuvant therapy. Frontline usual care in the trial consisted of watchful waiting plus self-care with local throat antiseptics and/or anaesthetics (solution, lozenges). Antibiotic medication, typically Amoxicillin tablets or liquids, and/or antipyretic and analgesic therapy for treatment of ATIs was prescribed by physicians for both adults and children as they deemed appropriate in both study groups. In addition to the physicians documenting the number of ATIs, based on their diagnosis, and mean infection free time that occurred during the trial period, as well as frequency in the use of antibiotics, study participants also kept diaries where they recorded impacts on their daily activities. The trial reported that the number of patients without an episode of ATI was significantly greater in the adjuvant therapy group that in the usual care alone group, 67.2% versus 37.5% (p < 0.0001). The hazard ratio for having at least one ATI in the adjuvant therapy group was 0.45 compared to the usual care alone group. There was also significantly less use of antibiotics to treat ATIs when they occurred in the adjuvant therapy versus the usual care alone group (37% vs 58.2%, p < 0.0008).

### Model structure

**Fig. 1:**
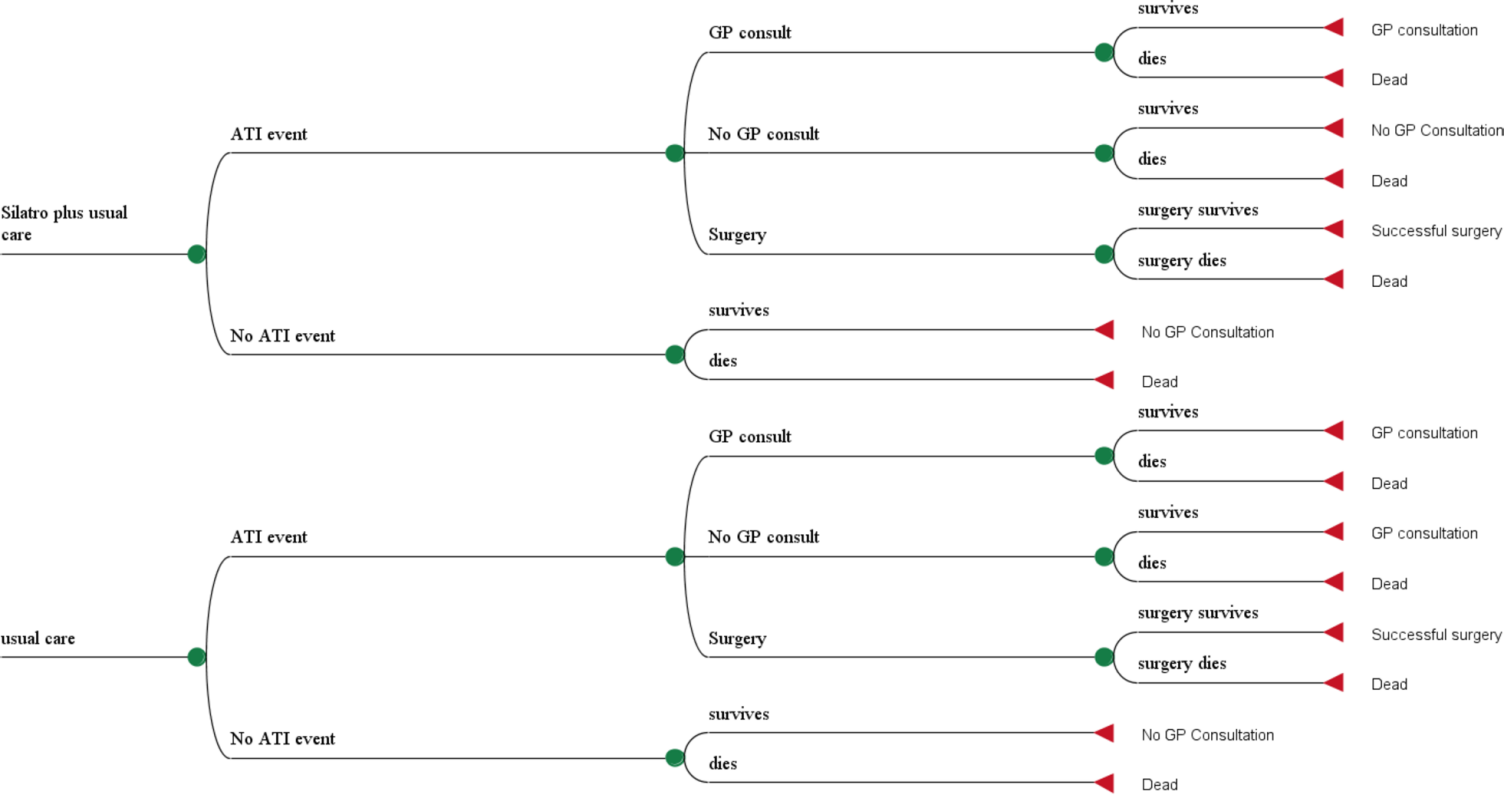
Excerpt of health economic model structure showing care pathways in real world practice in Germany starting with patients presenting to a primary care doctor (GP) with a potential acute throat infection (ATI)

We assumed that our Markov model would have 3 cycles per annum as in the effectiveness trial that we draw on there were 3 periods of active care of 8 weeks duration and 8 weeks subsequent observational follow up in 12 months. We chose a 2-year-time frame for the model, given that 2 ATIs in 2 consecutive years is one definition of recurrent ATI, and this has implications for potential future use of expensive tonsillectomy surgery. However, we report results from a one-year time frame perspective as well in sensitivity analysis. Our model reflects typical usual care pathways in Germany. In Germany, adults or children with a history of ATIs do or do not present to their primary care doctor (GP) in case of an ATI and if they suspect that they may have a further potential ATI. In case of their presentation, the GP then takes a patient history and performs a Centor assessment for adults or a McIsaac-adjusted-Centor score for children [19]. In our model therefore we assume that where Centor scores are at their highest levels of 3 or 4, in Germany, individuals will either receive usual care alone (consisting of watchful waiting/self-care with antiseptics and/or anaesthetics, plus discretionary use of antibiotics and/or analgesics/antipyretics for the treatment of ATIs) or they will receive a regimen of SilAtro-5-90 plus usual care (Fig. 1). The dosage regimens for adjuvant SilAtro-5-90 in each treatment cycle in the model are assumed to be the same as in the trial where each of the three 8-week active treatment cycles, consisted of 1 tablet 3 times per day for children (age 6 to < 12 years) and 2 tablets 3 times per day for adolescents and adults over 12 years. We assumed, as in the trial, that each 8 weeks treatment cycle would be followed by an 8 weeks period where only usual care would be provided.

**Fig. 2:**
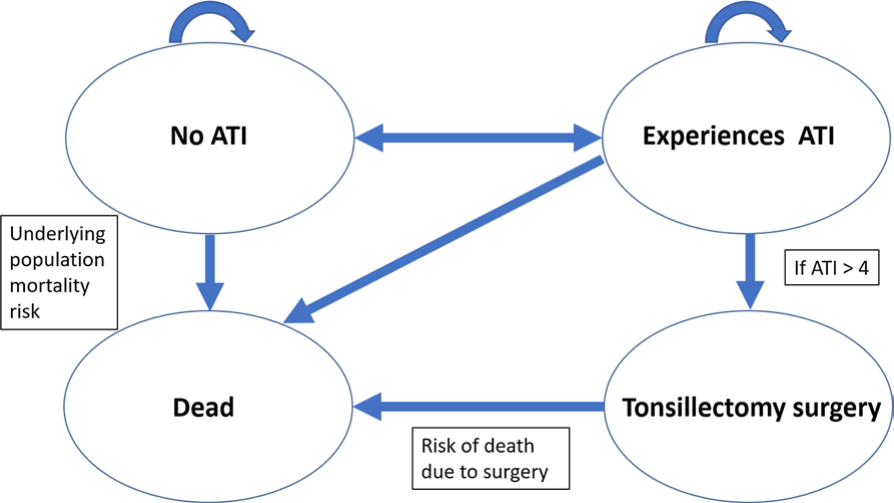
Structure of the Markov model showing the 4 possible transition states at the end of any cycle (*ATI* acute throat infection)

There are 4 possible transition states at the end of each cycle (Fig. 2). If there has been no ATI we assume that individuals may continue to self-medicate with SilAtro-5-90 as a preventive measure (as observed in the trial) in the next cycle, but with no further GP consultation. We assume that all individuals in both arms of the model who experience a recurrent ATI have a further GP consultation during each cycle in line with usual German care and they may potentially be prescribed a course of antibiotics. In line with current recommendations in Germany [20] our Markov model also assumes that tonsillectomy surgery may be offered as an alternative treatment option to patients who have had a minimum of 4 repeat ATIs events. In our model, we conservatively assume that successful tonsillectomy surgery eliminates the ATIs and the individual no longer needs further treatment. This is conservative as there can be a very small risk of reoperation. There is also a fourth death state, reflecting both general population mortality risk and the very small increased risk associated with tonsillectomy surgery. Mortality due directly to ATIs is extremely rare so we conservatively assume that there is no enhanced risk of mortality due to incidence of recurrent ATIs. We assume that these care pathways may continue into a second year, given the model population’s previous history of recurrent ATIs, and also mindful that moderate recurrent ATI can be defined as 2 ATIs in 2 consecutive years.

### Model parameters

Table 1 summarises parameters used in the model, assumptions on distributions used in probabilistic sensitivity analysis and sources for data. All analyses were modelled, implemented and performed with TreeAge Pro decision modelling software [21]. Expected rates of ATIs in the model for intervention and usual care groups were assumed to be the same as those reported in the multi-centre trial. The expected rate of ATI per cycle was assumed to be one third of the rate observed in the 12-month period. The likelihood of being treated with antibiotics following recurrent ATI is also assumed to be the same as that recorded in the trial. Where antibiotics are used, we assume, in line with typical German practice, that treatment lasts for 7 days, with a lower dosage for children (aged 6 to < 12 years) compared to adults and adolescents over 12 years. In line with the trial, we assume that patient age has no impact on the effectiveness of adjuvant therapy in reducing ATI rates. The mortality rate associated with tonsillectomy was based on registry data over 8 years in Sweden covering more than 82,500 procedures where just 2 deaths occurred [22]. Underlying 1-year both-sex probability of mortality was taken from German life tables for 2019 in the WHO’s Global Health Observatory Data Repository [23]. The probability of mortality per cycle was assumed to be one third of the annual mortality rate.

**Table 1:** Model parameters

Input parameter	Deterministic value	Values in one-way sensitivity and threshold analyses	Values in probabilistic sensitivity analyses	Assumption on distribution in probabilistic sensitivity analysis	Source
Costs					
SilAtro-5-90 costs per month (C)	€6.73	L: €3.37	–	Fixed	Palm et al.
		U: €10.10			
SilAtro-5-90 costs per month (AA)	€13.45	L: €6.73	–	Fixed	Palm et al.
		U: €20.18			
Tonsillectomy surgery (C)	€3495	L: €1748	–	Fixed	German Diagnosis Related Groups (DRG) data 2019
		U: €5243			
Tonsillectomy surgery Adolescents and Adults (AA)	€2538	L: €1269	–	Fixed	German Diagnosis Related Groups (DRG) data 2019
		U: €3807			
Antibiotics (1-week course) (C)	€12.41	L: €6.21	–	Fixed	German pharmacy data 2019
		U: €18.62			
Antibiotics (1-week course) (AA)	€13.09	L: €6.55	–	Fixed	German pharmacy data 2019
		U: €19.64			
Pharmaceutical dispensing fee	€5 per item for adults	–	–	Fixed	German pharmacy data 2019
General practitioner (per consultation)	€28	L: €14	–	Fixed	Data from the Association of Statutory Health Insurance Physicians 2019
		U: €42			
Daily productivity loss cost	€175.94	L: €87.97	α = 6.736	Gamma	Federal Office of Statistics 2019
		U: €263.91	λ = 0.038		
Travel time to/from GP	1.5 hours	L: 0.75 hours	–	Fixed	Assumption linked to mean travel distance
		U: 2.25 hours			
Travel time to/from hospital	3 hours	L: 1.5 hours	–	Fixed	Assumption linked to mean travel distance
		U: 4.5 hours			
Time out of role due to ATI per treatment cycle in intervention group	0.05 days^a^	L: 0.025	α = 1.082	Gamma	Palm et al.
		U: 0.075	λ = 20.945		
Time out of role due to ATI per treatment cycle in control group	0.16 days^a^	L: 0.08 days	α = 2.217	Gamma	Palm et al.
		U: 0.24 days	λ = 13.917		
Discount rate costs	3.0%	L: 0%	–	Fixed	Eunethta
		U: 6%			
Discount rate outcomes	3.0%	L: 0%	–	Fixed	Eunethta
		U: 6%			
Effectiveness and other parameters					
Number of ATIs in 12 months prior to baseline	2.5	L:2	Min.: 2	Pert	Palm et al.
		U:3.75	Max.: 3.75		
Probability of having an ATI in cycle with intervention	0.11	L: 0.055	α = 0.323	Beta	Palm et al.
		U: 0.165	β = 2.629		
Probability of having an ATI in cycle with usual care only	0.21	L: 0.105	α = 1.113	Beta	Palm et al.
		U: 0.315	β = 4.185		
Number of expected ATIs in cycle with intervention if patient has an ATI	0.54	L:0.27	α = 1.736	Gamma	Palm et al.
		U: 0.81	λ = 3.217		
Number of expected ATIs in cycle with usual care only if patient has an ATI	0.66	L: 0.33	α = 2.089	Gamma	Palm et al.
		U: 0.99	λ = 3.176		
Probability of ATI being treated with antibiotics in intervention group	0.37	L:0.185	α = 3.407	Beta	Palm et al.
		U: 0.555	β = 5.811		
Probability of ATI being treated with antibiotics for ATI in usual care only group	0.58	L: 0.29	α = 1.939	Beta	Palm et al.
		U:0.87	β = 1.392		
Probability of surgery if 4 or more ATIs per year (%)	0.25	L:0.125	–	Fixed	Authors’ assumption
		U:0.375			
Probability of all-cause mortality per cycle (%) (C)	0.000128452	L: 0.000064226	–	Fixed	WHO Global Health Observatory 2021
		U: 0.000192678			
Probability of all-cause mortality (%) per cycle (AA)	0.0007043578	L: 0.0003521789	α = 5.299	Beta	WHO Global Health Observatory 2021
		U: 0.0010565367	β = 7518.025		
Probability of mortality following tonsillectomy (%)	0.00242348	L: 0.001938784	α = 37.407	Beta	Østvoll et al. 2015
		U: 0.002908176	β = 15397.71		

*ATI* acute throat infection; *GP* general practitioner; *C* children aged 6 to < 12; *AA* adolescents and adults aged > 12 and up to 60; *L* lower; *U* upper

^a^Number of days reported as “standardized number of days” in Palm et al. i.e., number of days reported in the diary with “time out of role due to ATI” divided by the total number of diary days (ranging from 0 to 1, equivalent to a 0–100% scale)

Our analysis is conducted from a societal perspective. This takes account of costs within the health care system to health insurers, as well as any out of pocket costs to patients for medications, and days out of normal daily role due to recurrent ATIs, either work for adults or school for children. Costs of SilAtro-5-90 were based on over the counter average prices reported in the trial in Germany where a month’s course of SilAtro-5-90 cost €13.45 for adults and €6.73 for children (2019 prices). Resource use and costs of tonsillectomy surgery are taken from German Diagnosis Related Groups (DRG) data [24]. The costs of antibiotic treatment are taken from the mean pharmacy price across different products; for adults a course of penicillin treatment is €13.09 and for children aged 6–12 years this is €12.41 including a dispensing fee for pharmacists of €5 for each course of antibiotics for adults. The cost of a GP consultation according to data from the Association of Statutory Health Insurance Physicians is on average is €28.

We assume that there is a 2-week period out of normal role following surgery for adults and 1 week for children aged 6 to < 12 years. A mean hourly wage rate for all workers (full and part time) of €24.78 reported for 2019 by the German Federal Statistical Office (Destatis) was used to value hourly productivity losses. From this data, a day out of work was also assumed to have 7.1 hours [25]. We also include fixed out of pocket travel costs for each GP consultation. In the case of days out of school, we assume that there are additional informal care costs for one parent/guardian for each child under the age of 12 years who is unwell with an ATI. Impacts on days out of work or school were assumed to be the same as those reported in the multi-centre trial.

We assume very conservatively that all surgery for adults is day surgery without an overnight stay, but that for children aged 6–12 years the procedure involves an overnight stay. Typically, adults will on average take 2 weeks and children 1 week to recover from surgery. Making use of data reporting average travel distance to GPs of 2.97 km and hospitals of 13.25 km [26] we have assumed that travel time to and from each GP visit would be 1.5 hours while for hospitals this would be 3 hours. We have again valued this time using the mean hourly rate of €24.78 for all workers. We have not included any direct travel costs, e.g., for public transport or petrol.

All costs are reported in 2019 Euros and both costs and outcomes are discounted at an annual rate of 3.0% in the second year of the model [27]. All costs were sourced in 2019 Euros with the exception of intervention costs, which were uprated to 2019 prices using the Campbell-Cochrane Economics Methods Groups EPPI-Centre Cost Converter [28]. The model is run separately for children (aged 6–12 years) and adolescents and adults (aged over 12 years).

### Sensitivity analyses

A number of sensitivity analyses have been conducted to assess the robustness of results to underlying input parameters and assumptions. This includes univariate sensitivity analysis of specific model parameters, where the model is run varying the values of key parameters one at a time by 50% in either direction from their mean values in Table 1. We also identified threshold values of parameters at which the intervention became dominant or was dominated by usual care. In addition, we also conducted probabilistic sensitivity analysis using Monte Carlo simulation modelling to determine incremental cost effectiveness from 10,000 replications sampled from within parameter distributions.

## Results

In our model, for adults or children over the age of 12 years in our base case scenario from the societal perspective, the incremental cost per additional ATI avoided in the intervention group is €156.64. The total expected cost per patient over the 2-year time period of the model in the intervention group is €297.47 compared with €225.64 in the usual care only group. 0.46 additional ATIs would be avoided per patient in the intervention group over this time period. Most of these costs are out of pocket costs to patients, except for consultations with general practitioners and some of the costs of antibiotics, which would be borne by health insurers. As Table 2 indicates, some of the costs of intervention of €155.57 over 2 years are offset mainly by a reduction in GP consultations and the travel time of individuals to attend those consultations. There are greater productivity losses in the usual care group due to time out of normal daily role. While use of antibiotics is lower in the adjuvant therapy group the very low cost of antibiotics means that this only has a negligible impact on costs. In our base case scenario there are no costs for tonsillectomy surgery. This is because we assume on average each patient had a history of 2.5 ATIs on entering the model, while the number of expected additional events in the usual care only group is 0.92 ATIs compared with 0.46 ATIs in the intervention group. So, neither group ever reaches the point, i.e., 4 ATIs in a 2-year period, where surgery might be considered. When running the model for children aged 6–12 years we find that the adjuvant therapy plus usual care strategy is associated with both better outcomes and lower costs than care as usual. This is because the costs of intervention are lower for children due to lower dosage levels.

**Table 2:** Summary of cost effectiveness results based on a 2-year period

	Usual care plus SilAtro-5-90	Usual care only
Adults and adolescents over 12 years		
Adjuvant therapy	155.57	–
Antibiotics	1.67	6.16
GP consultations	58.91	84.57
GP—travel time	78.20	112.27
Tonsillectomy surgery	0.00	0.00
Tonsillectomy surgery—travel time	0.00	0.00
Productivity loss	3.13	22.65
Total cost (95% CI)	€297.47 (€220.67, €1403.94)	€225.64 (€72.83, €1905.03)
ATIs (95% CI)	0.46 (0.00, 2.22)	0.92 (0.00, 2.98)
ICER (€/ATI averted)	€156.64	
Children aged 6–12 years		
Adjuvant therapy	77.84	–
Antibiotics	1.67	6.16
GP consultations	58.91	84.57
GP—travel time	78.20	112.27
Tonsillectomy surgery	0.00	0.00
Tonsillectomy surgery—travel time	0.00	0.00
Informal care costs	3.13	22.65
Total cost (95% CI)	€219.75 (€142.99, €1,519.65)	€225.64 (€73.16, €2143.82)
ATIs (95% CI)	0.46 (0.00, 2.22)	0.92 (0.00, 2.95)
ICER (€/ATI averted)	Dominant (intervention has lower costs and better outcomes)	

*ICER* incremental cost effectiveness ratio (usual care plus SilAtro-5-90 vs. usual care); *CI* confidence interval; *ATI* acute throat infection; *GP* general practitioner

### Sensitivity analysis

#### One-way sensitivity and scenario analyses

**Fig. 3:**
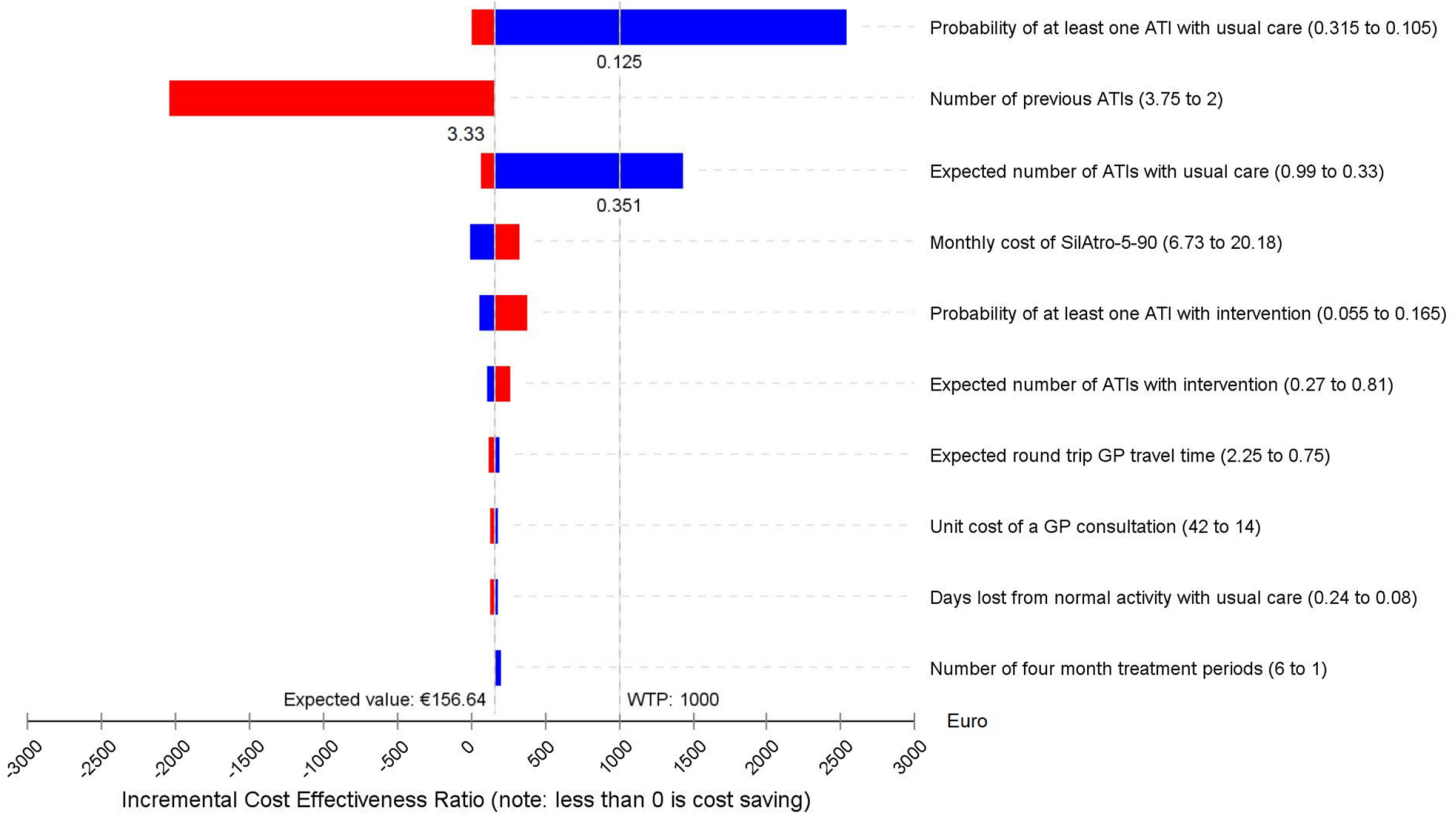
Tornado diagram showing most sensitive parameters in one way sensitivity analysis for adolescents and adults model (*WTP* willingness to pay; Numbers in the parenthesis show the upper and lower bounds of the model parameters used in sensitivity analysis in Table 1; Red segments of bars in the diagram indicate increasing values of each parameter and blue segments decreased values. Values to the right of our mean expected value of the ICER of €156.64 per ATI averted indicate a reduction in cost effectiveness of intervention relative to usual care, while those to the left indicate an improvement in cost effectiveness.)

The sensitivity of the cost effectiveness ratios for the adolescent and adult model to individual model parameters are shown as a ‘Tornado’ diagram in Figure 3. This figure shows the sensitivity of the results in hierarchical order for the ten parameters with the greatest sensitivity. The figure indicates that model results are most sensitive to changes in the probability of ATI in the usual care group. If the probability of at least one ATI after usual care falls by 50% then the cost per ATI averted increases from €157 to €2533. If the probability of ATI after usual care fell by 43% from 0.21 to 0.12 then the cost per ATI averted would be above the illustrative willingness to pay threshold of €1000 threshold shown in Figure 3. Patient history on entering the model also has an impact. If the number of ATIs prior to entering the model increases to above 3.33 then the intervention becomes cost saving. This is because there is then a chance of having more than 4 ATIs, triggering the possibility of tonsillectomy surgery. Varying the number of treatment cycles also has an impact on cost effectiveness. With just one 4-month cycle of treatment the cost per ATI averted would be €204.19 compared to our base case of €157 per ATI averted with 6 cycles of treatment. If our Markov model only had 3 treatment cycles in a 1-year period then the cost per ATI averted would be slightly higher at €166. If the expected number of ATIs with usual care decreases by 50% then the cost per ATI averted increases to €1435, while a 50% increase would reduce the cost per ATI averted to €66. A 47% decrease in expected number of ATIs in the usual care group would also mean the notional willingness to pay threshold would be crossed. Figure 3 also indicates that the model does not appear very sensitive to all other parameters, including the costs of SilAtro-5-90 and days out of role due to ATI. Parameters such as the probability of receiving antibiotics following ATI, likelihood of receiving surgery after 4 ATIs, days lost from productivity with intervention, unit costs of tonsillectomy surgery, travel time to and from hospital, as well as all-cause mortality and tonsillectomy-surgery related mortality have a negligible impact on the incremental cost effectiveness ratio and are not shown. We have not shown the Tornado diagram for children as a very similar pattern was seen. Most notably if there was a 50% reduction of the probability of at least one ATI in the usual care group the intervention would no longer be cost saving, but instead would have a cost per ATI averted of €1208. Intervention would continue to dominate usual care if the model only ran for 1 year, if there were just one 4-months model cycle it would have a cost per ATI averted of €34.59.

In addition to varying parameters by 50% in either direction, we also identified 5 parameters where there were thresholds at which the intervention would become dominant or be dominated compared to usual care. These are shown in Table 3. The threshold for past history of ATIs for adults and adolescents where the intervention dominates (has lower costs and better outcomes) is 3.33 ATIs. For the model for children, even without any past ATIs, the reduction in expected number of ATIs means that the intervention is always dominant. For children the intervention group no longer dominates usual care when the probability of ATI increases to 0.118, equivalent to a 42% reduction in ATI. For children only, the intervention is cost saving until the expected number of ATIs in children experiencing ATI in any cycle reduces from 0.66 to 0.52. For children if the model only had 2 treatment cycles, covering an 8 month-time period rather than 2 years then the intervention would no longer dominate usual care. Finally, for both adults and children if the cost of SilAtro-5-90 adjuvant therapy was below €14.49 then the intervention would be dominant. Compared to base case, for the intervention to be dominant for adults and adolescents would mean the costs of adjuvant therapy would have to fall by 46%.

**Table 3:** Threshold analysis

Parameter description	Adolescents/adults	Child aged 6–12
Number of recurrent ATIs in 12 months prior to treatment (base case 2.5)	3.33 ATIs: intervention becomes dominant	No threshold. Intervention dominant in all scenarios
Probability of ATI after intervention	No threshold	Intervention no longer dominates where probability of ATI rises to 0.118
Reducing the expected number of ATIs in the control group in any one cycle (base case 0.66)	No threshold	Intervention no longer dominates when expected number of ATIs falls to 0.52
Number of treatment cycles (base case 6)	No threshold	Intervention no longer dominates when 2 or fewer treatment cycles
Cost of SilAtro-5-90 (base case €13 and €26 per cycle for children and adults)	Intervention becomes dominant when cost falls to €14.48	Intervention no longer dominates when costs increase to €14.49

*ATI* acute throat infection

#### Probabilistic sensitivity analysis

**Fig. 4:**
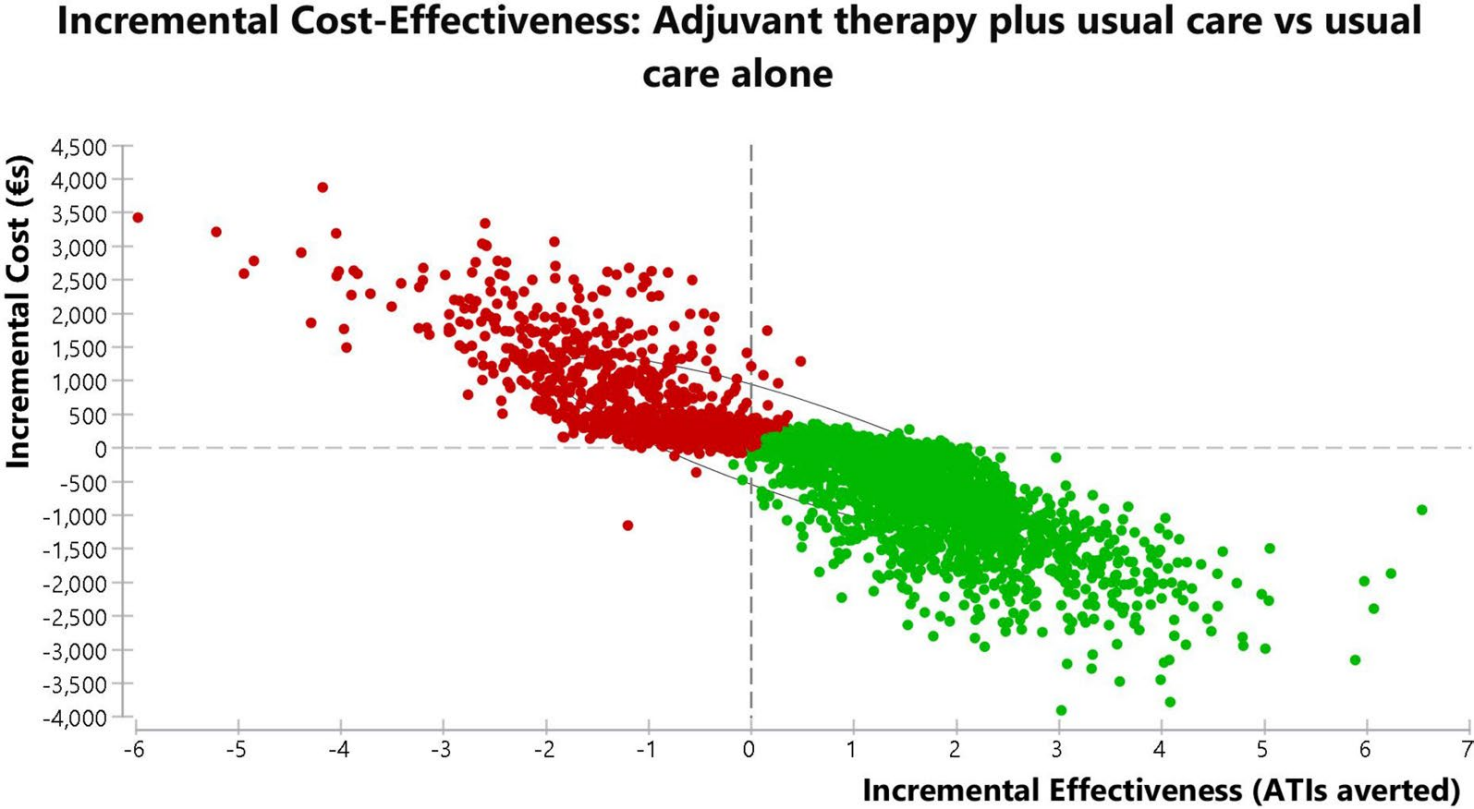
Cost effectiveness plane of the adults and adolescent model for incremental cost effectiveness of usual care plus adjuvant therapy versus usual care alone. The elliptical circle includes 95% of the simulations in the model (*ATI* acute throat infection)

**Fig. 5:**
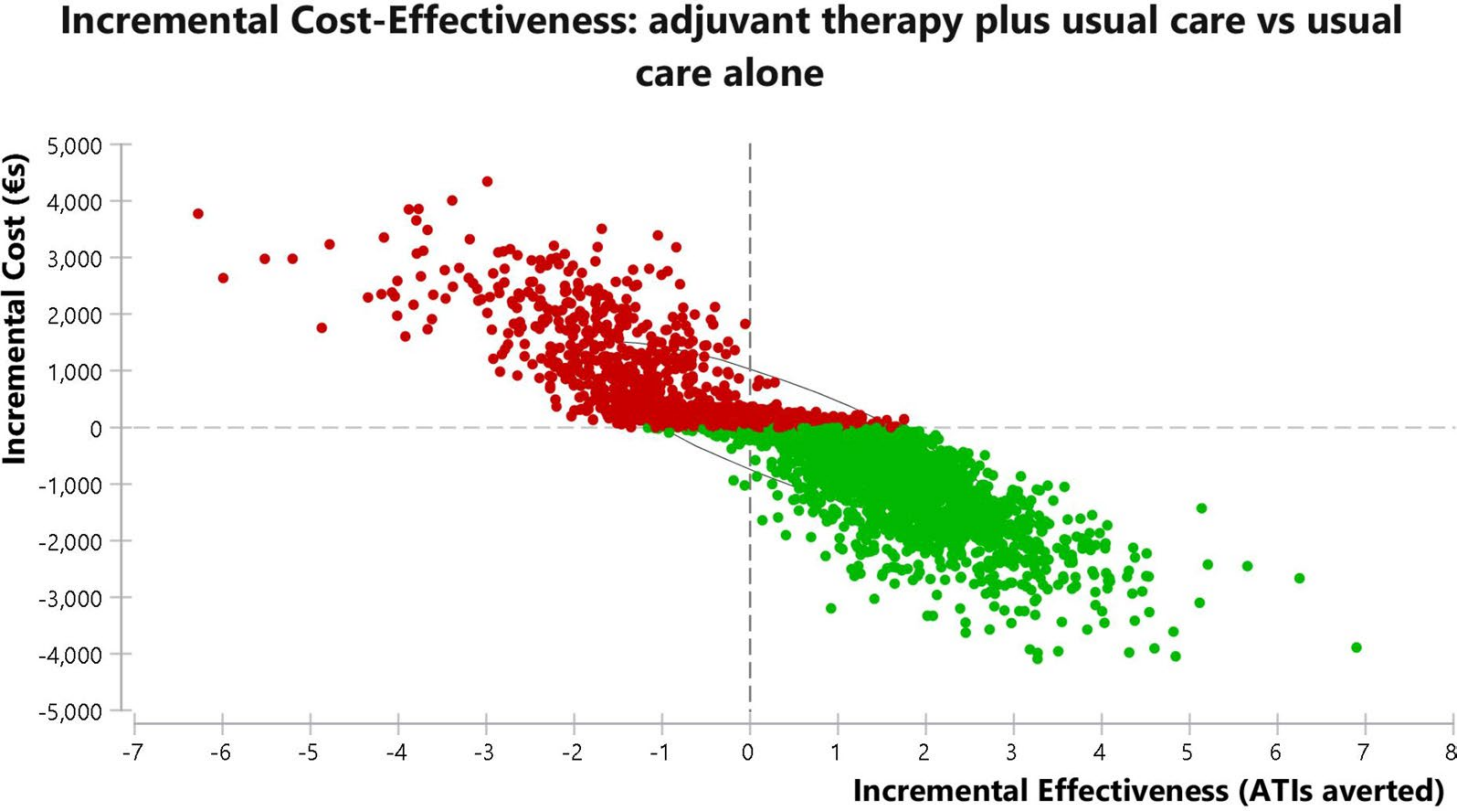
Cost effectiveness plane of the children model for incremental cost effectiveness of usual care plus adjuvant therapy versus usual care alone. The elliptical circle includes 95% of the simulations in the model (*ATI* acute throat infection)

Probabilistic sensitivity analysis was also performed, using Monte Carlo simulation with 10,000 replications sampled from the distributions for adults presented in Table 2. The cost effectiveness plane for the adults and adolescent model for incremental cost effectiveness of usual care plus adjuvant therapy versus usual care only is shown in Fig. 4, while the results for the model for children are given in Fig. 5.

For adults and adolescents, the distribution of the simulations indicates that SilAtro-5-90 adjuvant therapy in addition to usual care would avert ATIs at lower costs in 3157 (32%) of simulations. In a further 4493 (45%) of simulations adjuvant therapy would avert ATIs but at higher cost. Society would then have to consider whether it would be willing to pay these extra costs for the ATI averted. This is due to the extra costs of surgery incurred by some individuals who experience more than 4 ATIs in the model. The simulation also suggests there is a 23% chance that the intervention would be inferior to usual care only with higher costs and fewer ATIs averted. When running probabilistic sensitivity analysis on the model for children aged 6–12 years, from a societal perspective in 4996 simulations (50%) adjuvant therapy plus usual care is cost saving with additional ATI events averted at lower cost. In another 2615 simulations (26%) intervention plus usual care is more effective but also more expensive than usual care. The simulation model suggests there is a 23% chance that the intervention would be inferior to control with higher costs and fewer ATIs averted.

**Fig. 6:**
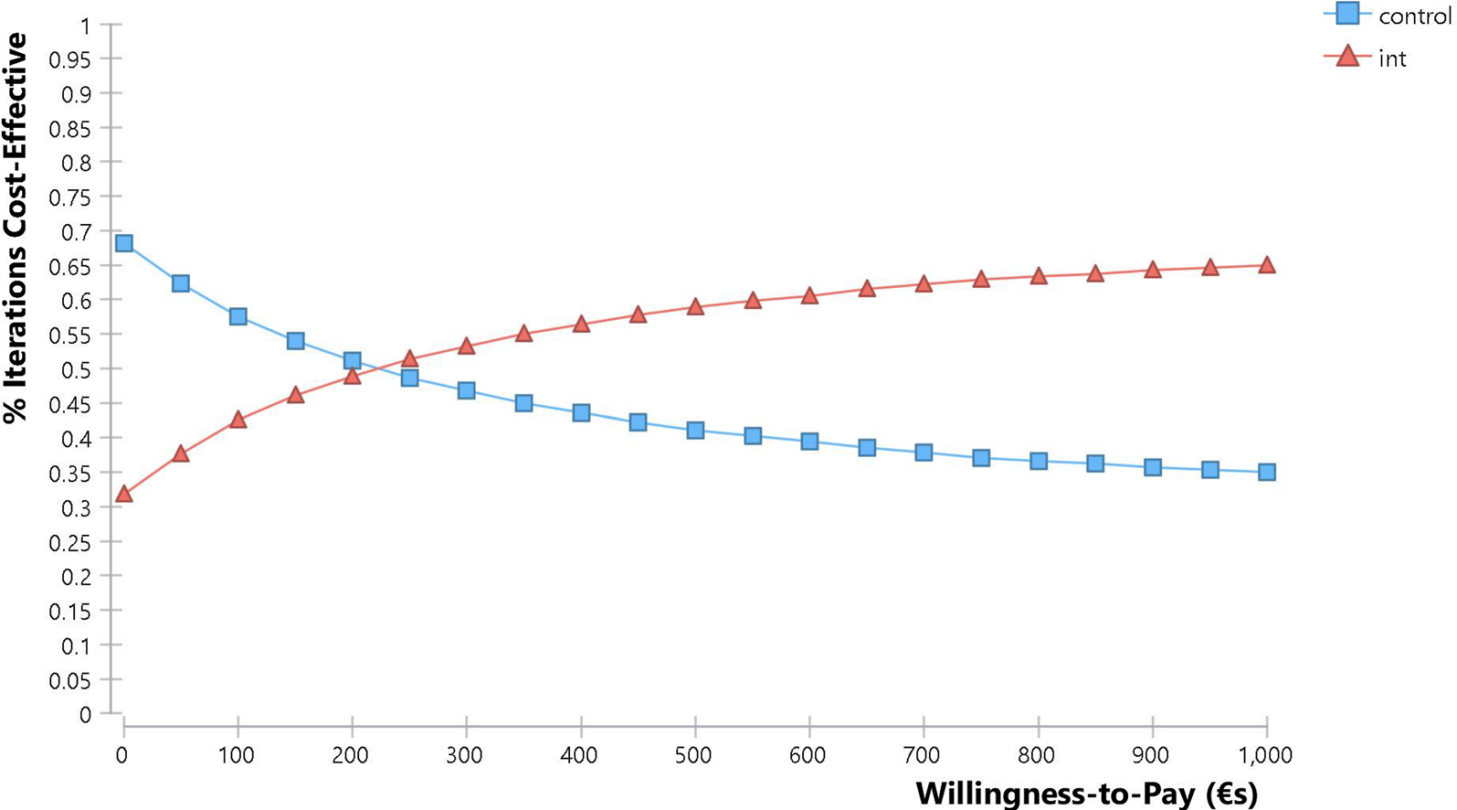
Cost effectiveness acceptability curve: adolescents and adults

**Fig. 7:**
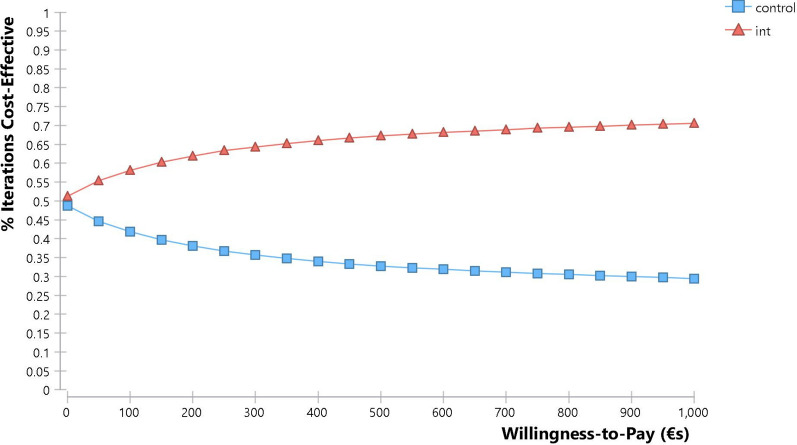
Cost effectiveness acceptability curve: children

We also constructed cost effectiveness acceptability curves for both of these models based on the probabilistic sensitivity analyses. These curves indicate the likelihood that an intervention is cost effective at different willingness to pay levels. For adults and adolescents this indicates that for a willingness to pay of more than €230 per ATI averted adjuvant therapy plus usual care has a greater chance of being cost effective than usual care, with this rising to a 65% chance of being cost effective at a hypothetical willingness to pay level of €1000 (Fig. 6). For children, even if their parents/guardians are unwilling to pay anything per ATI averted, adjuvant therapy plus usual care still has a 51% chance of being cost effective, with this rising to a 71% chance of being cost effective at a hypothetical willingness to pay threshold of €1000 (Fig. 7).

## Discussion

Tonsillitis is a common problem in the primary care sector, which in cases of a bacterial origin is treated with antibiotics. For an integrative patient care approach, standardised, safe, and cost-effective treatment strategies are essential. While several authors have dealt with the question of cost-effectiveness of tonsillitis treatment, in particular concerning tonsillectomy [29, 30], integrative approaches such as homeopathy so far have not been analysed with respect to cost-effectiveness in tonsillitis, although there is a growing demand to conduct cost effectiveness studies in the field of Integrative medicine to inform decision making [31].

The present study for the first time analysed the cost-effectiveness of SilAtro-5-90 as an adjuvant therapy to usual care for patients with mild to moderate acute or recurrent tonsillitis based on a previously published trial. Our findings suggest that the intervention in our base case scenario indicates a cost of €156.64 per ATI averted. Policy makers need to think of how much they would be willing to pay to avert ATIs; at minimum, our model suggests for adults this must be more than €230 to be more favourable than usual care. In the case of children aged 6 to < 12 years, from a societal perspective adjuvant therapy appears cost saving with both better outcomes and lower costs than usual care only. In the case of children in half of the Monte Carlo simulations, it was shown that SilAtro-5-90 therapy averts ATIs at lower costs.

Our analyses also suggest that the more accurately the use of adjuvant therapy can be targeted at people with a history of recurrent tonsillitis, the more the cost effectiveness of the intervention is strengthened, as intervention may potentially help avoid surgical intervention. We therefore examined how differences in previous history of recurrent tonsillitis impact on the cost effectiveness of the intervention. In our base case scenario no individuals would meet the criteria for tonsillectomy surgery as no individuals would experience 4 or more ATIs in 2 years. However if intervention were targeted to those with a higher level of past ATI then there are additional potential benefits if tonsillectomy surgery can be avoided. In our sensitivity analysis, where we assume that there would be a 25% chance of being referred for tonsillectomy surgery after 4 recurrent events, if an individual has on average a history of 3.33 events prior to entering the model (rather than our expected average of 2.5 prior ATIs) then the intervention group will have both lower costs and better outcomes than the care as usual group. In the multi-country trial of SilAtro-5-90 20% of individuals entered the study with a history of 4 or more ATIs [16]. For this type of patient, SilAtro-5-90 should be considered as a promising adjuvant treatment option as it may help contribute to the chance of avoiding expensive tonsillectomy surgery, which is the most common conservative treatment option, in case of frequent episodes of ATIs. While surgery is generally successful, each surgery bears a small risk of pain and side effects, as well as mortality, all of which could have a negative impact on society in terms of loss of productivity and costs for the surgery.

Besides benefits to individual patients of having fewer ATIs and in the long term potentially avoiding surgery, there are potential spill-over benefits beyond the patient to the health care system and society as every ATI averted reduces the use of antibiotics, helping to address the threat of AMR. These benefits are not captured in our economic model. All of these potential advantages depend on patients making use of the intervention. The cost of SilAtro-5-90 therapy is borne by patients rather than health insurers as homeopathic medicines are over the counter medications. Ultimately, patients will need to make a judgement as to whether they are prepared to pay €13.45 (adults) or €6.73 (children) per month of treatment for the additional ATIs averted. The costs appear relatively modest as they fall well within reported expenditures for homeopathic remedies (ranging from €3.70 to €124.54 per month) [8], although as with all out of pocket medications there will be inequalities in access and uptake linked to levels of income.

## Limitations

Many of our Markov model parameters are drawn from a single multi-county open label trial where participants were only followed up for 12 months. The method of the clinical trial is clearly described in the original publication [16], the study diagnoses were medically confirmed, and the robustness of the results were underlined by sensitivity analysis. To date, however, the clinical trial has not been repeated, nor has the mechanism of action of SilAtro-5-90 been researched. While we have undertaken extensive sensitivity analyses to mitigate for uncertainty in model parameters, more trials are needed to see if these results from a single study can be replicated, both in trials and in pragmatic studies in real world conditions. Moreover, differences in health system structures, attitudes towards complementary therapies and real-world practice between countries, as potentially patterns of effectiveness and antibiotic prescribing behaviour may vary across settings.

Another limitation is a lack of separate published data on the ATIs averted in children aged under 12 years compared to impacts in adults and adolescents. The published trial paper indicated that age did not have a significant impact on outcomes so we have assumed that effectiveness rates are the same regardless of age. Nevertheless, there might be differences in effect that we are unaware of; potentially there might also be differences by gender. Future trials need to assess the effectiveness and cost-effectiveness of adjuvant therapy for different age groups, genders and other population groups in different country settings. If economic analyses can be conducted prospectively alongside trials so that there is access to individual patient level data future studies might also consider looking at longer term cost effectiveness using discrete event simulation modelling with time to ATI event rather than the likelihood of experiencing an ATI event being the primary outcome. There is also a need to assess whether the uptake and sustained use of adjuvant therapy outside of the conditions of a controlled trial can be replicated in real-world settings, particularly as patients would have to pay out-of-pocket for adjuvant therapy. This is also something that could be considered in future implementation studies in Germany and other settings.

Another limitation is the lack of published data on quality of life associated with ATIs using a validated quality of life scale such as the EUROQOL EQ-5D that is used in health economic analysis [32, 33] or even a specific assessment instrument such as the Sore Throat Quality of Life Questionnaire (STQoL) [34] to get a more differentiated picture of the impact of the intervention. We have noted that there is no accepted willingness to pay threshold for the avoidance of ATIs. If we had EQ-5D quality of life data we could have calculated the cost per QALY gained. Established cost effectiveness thresholds could then have been used; this would have allowed comparisons to be made between investment in different measures not only to tackle ATI, but any other potential use of health care resources. We also considered using published utility estimates associated with ATIs to estimate QALY gains to overcome this limitation, but were unable to source any utility values for ATIs suggesting that there have been very few economic evaluations for interventions to reduce ATIs (other than evaluations of tonsillectomy) [29]. We are however aware of one trial and economic evaluation completed in 2020, but as yet unpublished, of tonsillectomy in adults in the UK that has collected quality of life data on ATIs; this potentially will provide utility values to inform future modelling studies [35]. In saying that, one of the challenges, even with the use of QALYs thresholds is that they reflect the willingness of health care funders to cover the costs of any treatment. However, in this case much of the immediate cost and cost offsets associated with treatment or usual care are borne by patients and their families rather than health care systems.

Our model estimates of economic impact may however be conservative; we have not included any costs for hospitalization for acute tonsillitis, nor did we include the costs of complications associated with surgery, with a Canadian study indicating that 3% of children are readmitted within 30 days [36]. In the model, we have assumed that all tonsillectomies are day procedures for adults and have just one night’s stay for children; procedures can still be performed on an inpatient basis, with several days spent in hospital. For children we have also conservatively assumed that only one parent gives up time for their usual activities to accompany their child to any surgical procedure, yet 64% of children in a previous Belgian analysis for 275 children who underwent tonsillectomy were accompanied by both parents [30]. While this would not affect our base case findings, as no individuals would meet the criteria for tonsillectomy surgery, it would affect the likelihood of being cost effective in our probabilistic sensitivity analysis.

With respect to the treatment, our analyses are also limited by the fact that only the cost scenario for penicillin V was calculated. We have not considered the impact of penicillin allergies where other, possibly more expensive antibiotics would then be necessary (e.g., Macrolides like Clindamycin). The same limitation is given for surgical procedures: tonsillectomy was assumed for surgery, while other procedures like tonsillotomy, albeit far less common in adults and mostly applied in children, have not been considered as a surgical procedure in our analysis.

### Integrative medicine and cost effectiveness

Papers often claim that integrative medicine is potentially cost-effective compared to conventional treatment [37, 38]. Even if evidence for this claim is still weak, particularly in the field of homeopathy [39], some encouraging results have recently been published. In a prospective observational study, add-on homeopathic treatment in routine care seemed to be cost effective from an insurer perspective in terms of additional costs per quality-adjusted life year for 3 out of the 5 assessed chronic diseases: migraine or headache, atopic dermatitis and depression [40]. From a health services perspective, a systematic review of economic evaluations found one study that according to the authors compared “usual care to usual care plus access to multiple licensed CIM practitioners” [41]. In this review, the authors thus recommend that further studies should at least include one arm of usual care and should contain sensitivity analyses. Finally, they state that “more consideration be given to modelling as a method to estimate economic outcomes for existing effectiveness trial results, to increase the reliability of results”. The present economic analysis fully takes up these recommendations and presents a full economic analysis in the field of primary care which is the major setting of homeopathy.

## Conclusions

This paper provides a real-world health economic picture of homeopathic treatment of recurrent tonsillitis in primary care indicating the importance of considering homeopathic treatment for this indication from a socio-economic perspective. It also underlines the possible role homeopathy can play as adjuvant therapy in the “no-antibiotics” or “delayed antibiotics” strategy for the treatment of RTI, thereby contributing to combating AMR. Additionally, the treatment with SilAtro-5-90 can avoid tonsillectomy with its inconveniences for patients due to surgery and costs for society (direct for the surgery and indirect, for e.g., absence of work). Further studies should take up this approach for a deeper understanding of potential fields of cost effectiveness, not limited to homeopathic primary care but for the full spectrum of integrative primary care.

## Supplementary Information

**Additional file 1.** Consolidated Health Economic Evaluation Reporting Standards – CHEERS Checklist.

## Data Availability

The datasets generated and/or analysed during the current study are available from the corresponding author on reasonable request.
